# Immunoenhancing effects of Gynostemma Pentaphyllum Extract on mucosal immunity against porcine epidemic diarrhea virus

**DOI:** 10.3389/fvets.2025.1636663

**Published:** 2025-08-22

**Authors:** Yanxiang Zhang, Ruihan Tang, Yuanqing Liu, Zhihui Hao, Kai Fan, Shanshan Xie, Bo Tang, Shuaiyu Wang

**Affiliations:** 1 College of Veterinary Medicine, China Agricultural University, Beijing, China; 2 Agricultural College, Hubei Three Gorges Polytechnic, Yichang, China; 3 Innovation Center of Chinese Veterinary Medicine, China Agricultural University, Beijing, China; 4 Key Biology Laboratory of Chinese Veterinary Medicine, Ministry of Agriculture and Rural Affairs, Beijing, China; 5 College of Veterinary Medicine, Henan Agricultural University, Zhengzhou, China; 6 Beijing Biopharmaceutical Technology Center, Zhaofenghua Biotechnology Co., Ltd. (Nanjing), Beijing, China

**Keywords:** Gynostemma Pentaphyllum Extract, porcine epidemic diarrhea virus, mucosal immunity, antibody level, veterinary vaccine

## Abstract

**Introduction:**

This study investigated the mucosal immunoadjuvant effects of *Gynostemma pentaphyllum* Extract (Gynostemma P.E), the bioactive constituents of Gynostemma pentaphyllum, against porcine epidemic diarrhea virus (PEDV).

**Methods:**

Twenty-four mice were randomly divided into four groups: a negative control group (intranasal administration of antigen only), a Gynostemma P.E-antigen mixture test group (intranasal administration), and two positive control groups (intramuscular injection of antigen or inactivated homemade vaccine, respectively). Fourteen days post booster immunization, spleen samples were collected to assess splenic lymphocyte proliferation activity. Intestinal segments were harvested for histological evaluation; duodenal intraepithelial lymphocytes (IELs) and IgA-positive cell numbers were quantified via H&E staining and immunohistochemistry (IHC). Serum and mucosal lavage fluid were analyzed for specific IgG and secretory IgA (sIgA) antibody levels.

**Results:**

The results demonstrated that Gynostemma P.E significantly promoted immune organ development, enhanced splenic lymphocyte proliferation (*p* < 0.05), and elevated serum IgG and nasal mucosal sIgA antibody levels (*p* < 0.05).

**Discussion:**

This study presents an innovative approach that integrates bioactive compounds from traditional Chinese medicine with intranasal mucosal immunization, offering new perspectives for combating gastrointestinal infections in veterinary medicine, and demonstrates that Gynostemma P.E significantly enhance PEDV-specific mucosal immunity in mice, providing a foundation for developing safer and more effective PEDV vaccines (and other veterinary vaccines), data supporting Gynostemma P.E as mucosal immunoadjuvants, and a theoretical framework for future clinical trials.

## 1 Introduction

Porcine epidemic diarrhea (PED), caused by porcine epidemic diarrhea virus (PEDV), is an acute enteric disease affecting pigs of all ages (1). The disease is particularly devastating to neonatal piglets, with mortality rates reaching 80–100% (2). Although inactivated and live-attenuated vaccines are currently available, effective control remains challenging due to PEDV’s high mutation rate, frequent co-infections with other pathogens, and potential recombination with transmissible gastroenteritis virus (TGEV) (3). These factors contribute to its persistent threat to China’s swine industry.

PEDV transmission occurs primarily through fecal-oral routes, indirect contact with contaminated fomites, and fecal-nasal exposure (4), all of which involve mucosal invasion. Therefore, enhancing mucosal immunity represents a critical strategy for infection prevention. While numerous mucosal adjuvants exist, few simultaneously meet the following essential criteria: (1) high safety profile, (2) capacity to induce potent immune responses with long-lasting memory, and (3) stability under varied storage conditions. Active components from traditional Chinese medicine have demonstrated significant immunomodulatory effects with excellent safety and stability profiles (5, 6), making them ideal candidates for immune modulation. Their prospects as adjuvants to enhance both the magnitude and durability of influenza vaccine responses are highly promising (7).

*Gynostemma pentaphyllum* is a traditional Chinese medicinal herb with a broad pharmacological profile. Previous studies have shown that both its total saponins and polysaccharides exert antiviral and immunopotentiating effects in animals, and evidence has indicated that gypenosides possess adjuvant activity in modulating cellular and humoral immunity (8–10). Recent work has also demonstrated the immune-enhancing effects of ginseng stem-leaf saponins against PEDV (11), yet no reports have described the use of intranasal gypenosides to boost intestinal mucosal immunity against PED.

Starting from the goal of “how to elevate intestinal mucosal immunity in swine herds,” the present study selected Gynostemma P.E as the test agent. Mice were intranasally immunized with inactivated PEDV antigen combined with Gynostemma P.E, and the impact on mucosal immunity was assessed. Measurements of nasal wash IgA levels, splenic lymphocyte proliferation, and intestinal mucosal immune parameters preliminarily confirmed that intranasal co-delivery of Gynostemma P.E with antigen significantly enhanced PEDV-specific mucosal responses. These findings offer a reference for improving the efficacy of PED and other veterinary vaccines and provide an experimental basis for the clinical application of Gynostemma P.E.

## 2 Materials and methods

### 2.1 Instruments, drugs, and reagents

The following equipment was used: MJ-78A autoclave (Shanghai Shiduke Instrument Equipment Co., Ltd), BBXW-20 ice maker (Beijing Boxiang Xingwang Technology Co., Ltd), D3024R centrifuge (SCILOGEX), microplate reader and vortex mixer (BIO-RAD). Gynostemma P.E (Catalog No. E3343) was purchased from Selleck Chemicals (United States). PED inactivated antigen and inactivated vaccine (inactivated antigen mixed with ISA 201 adjuvant at a 9:11 volume ratio) were kindly provided by Zhaofenghua (Nanjing) Biological Technology Co., Ltd. Mouse PEDV-IgG and IgA ELISA kits were obtained from Shanghai Jianglai Biotechnology Co., Ltd. CCK8 kit and DAB chromogen were purchased from Beijing Solarbio Science & Technology Co., Ltd. Goat anti-mouse IgA polyclonal antibody and HRP-conjugated rabbit anti-goat IgG antibody were acquired from Abcam (UK).

### 2.2 Preparation of Gynostemma P.E and vaccine

The purchased Gynostemma P.E (powder form, derived from the whole plant of *Gynostemma pentaphyllum*) was homogenized with inactivated antigen in phosphate-buffered saline (PBS) to formulate a vaccine solution. This solution contained 1 μL of antigen and 100 μg of the botanical extract per mouse, with a final immunization volume of 10 μL per mouse, and was freshly prepared before each administration.

### 2.3 Experimental animals and group immunization

Twenty-four healthy male Balb/c mice (6–8 weeks old, 20 ± 2 g) were purchased from Vital River Laboratory Animal Technology Co., Ltd. [Production license: SYXK (Jing) 2019–0025]. After 7 days of acclimatization, mice were randomly divided into 4 groups (*n* = 6): Group A (negative control) received a mixture of normal saline and inactivated antigen via intranasal administration. Group B (Gynostemma P.E + Ag) received a mixture of Gynostemma P.E and inactivated antigen via intranasal administration. Group C (Positive Control 1) and Group D (Positive Control 2) also received PED inactivated antigen and PED inactivated vaccine (containing ISA 201 adjuvant) respectively via intramuscular injection. All mice were immunized through the above routes on days 1 and 21. Each immunization delivered 100 μL of inactivated antigen with TCID₅₀ of 10^7.5^ per mouse. Samples were collected 14 days after the second immunization.

### 2.4 Sample collection and processing

Given that preliminary experiments showed no significantly higher antibody levels in any test groups compared to the control group two weeks after the first immunization, and that the extract began to exert adjuvant effects two weeks after the second immunization, serum and mucosal lavage samples were collected two weeks after the second immunization in studies using Gynostemma P.E as an adjuvant.

Serum samples were obtained via retro-orbital plexus puncture or enucleation. Mice were sacrificed by cervical dislocation, and nasal lavage fluid was collected by injecting ice-cold PBS into the trachea (100 μL collected from nostrils). Duodenal mucosa was homogenized and centrifuged. Thymus and spleen were weighed, and intestinal segments (5 cm) were either fixed in 4% paraformaldehyde or flash-frozen in liquid nitrogen for storage at −80 °C.

### 2.5 Specific IgG and IgA antibody titers measurement

Serum IgG and mucosal IgA levels in nasal lavage/intestinal homogenate were determined by ELISA. Absorbance at 450 nm was measured using a microplate reader, with antibody concentrations calculated against standard curves.

### 2.6 Splenocyte proliferation assay

Splenocytes were isolated by mechanical dissociation, filtration and centrifugation. Cells (3 replicates/sample) were cultured with ConA in 96-well plates, and proliferation was assessed using CCK8 kit. Stimulation Index (SI) was calculated as: SI = OD ConA-treated/OD untreated (12).

### 2.7 Histological analysis

Intestinal segments were paraffin-embedded, sectioned, and stained with hematoxylin–eosin (HE). Intraepithelial lymphocytes (IELs) were counted under light microscopy (10 villi/sample, scoring system: 0 = none; 1 = 1–5; 2 = 6–10; 3 = 11–15; 4 = 16–20; 5 > 20 cells/villus). For immunohistochemistry (IHC), sections were dewaxed, incubated with HRP-conjugated secondary antibody, developed with DAB, and counterstained with hematoxylin.

### 2.8 Statistical analysis

All data are presented as mean ± SD. Statistical analysis was performed using Excel and GraphPad Prism. *p* < 0.05 was considered statistically significant, while *p* < 0.01 indicated highly significant differences.

## 3 Results

### 3.1 Increased serum IgG and nasal mucosal sIgA antibodies by gypenoside treatment

**Figure 1:**
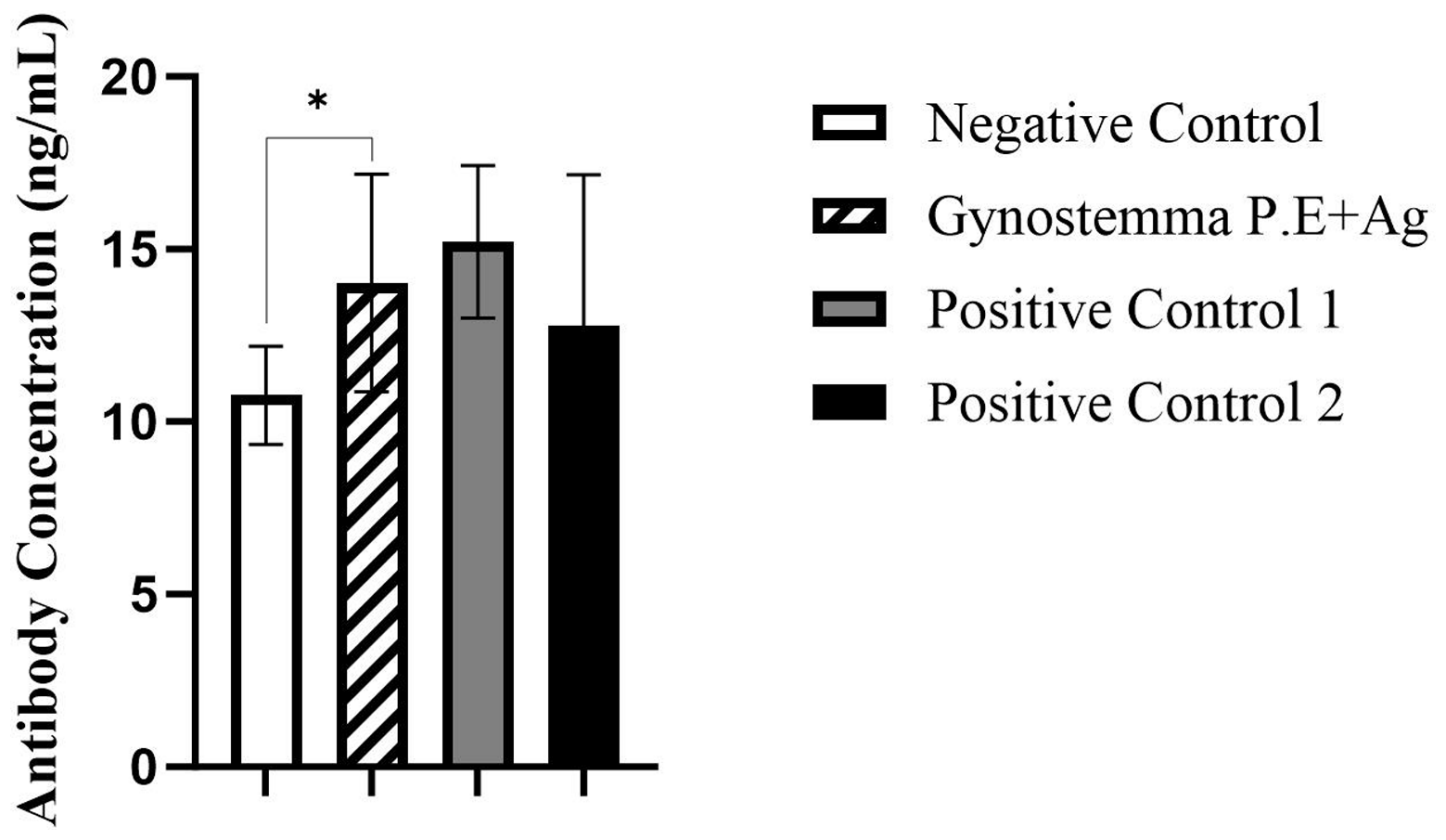
Serum IgG antibody levels. * indicates a statistically significant difference between groups (*p* < 0.05). Negative control: intranasal saline treatment; positive control 1: antigen injection group; positive control 2: vaccine injection group.

**Figure 2:**
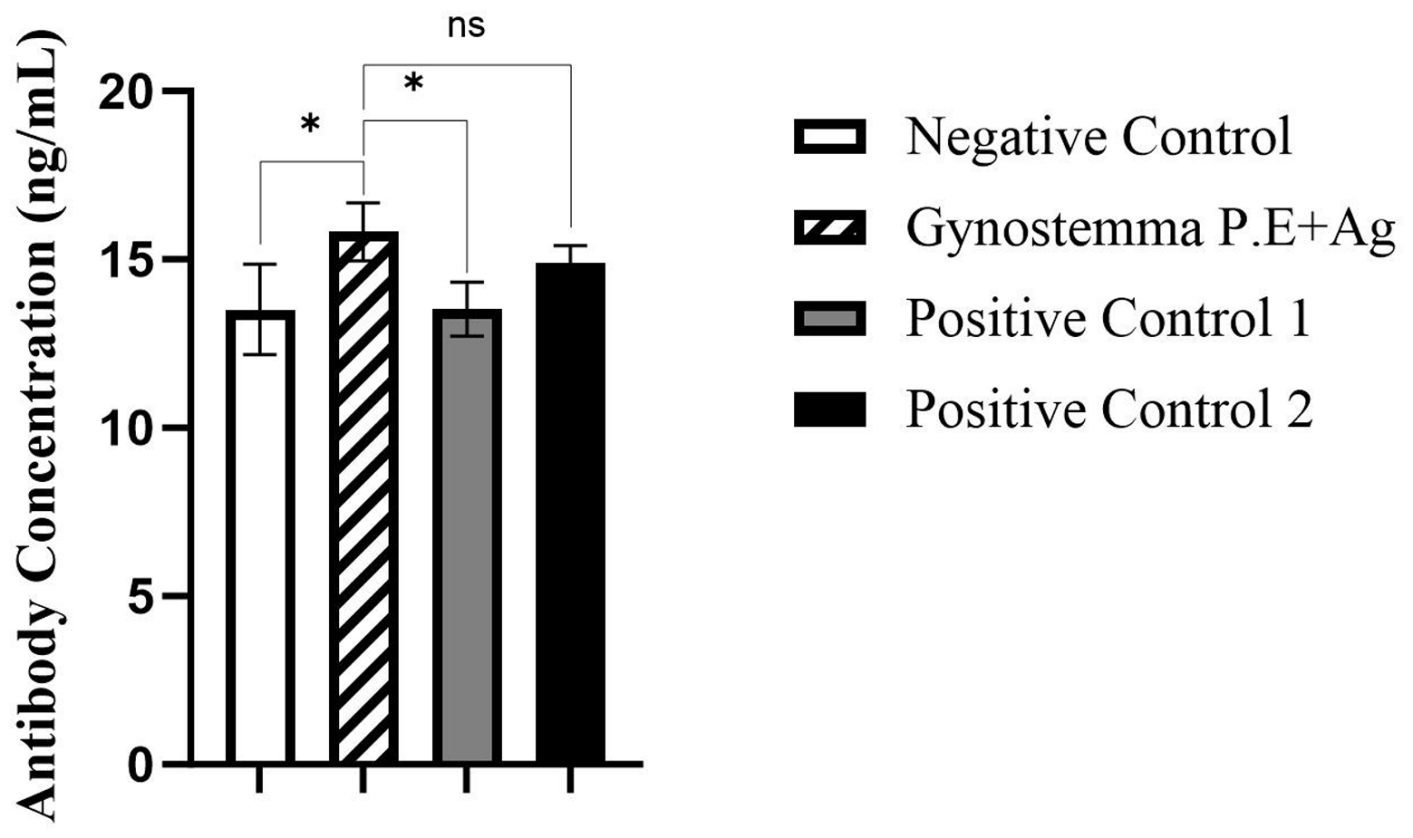
Detection of IgA antibody levels in nasal lavage fluid. * indicates a statistically significant difference between groups (*p* < 0.05); ns indicates no significant difference (*p* > 0.05).

**Figure 3:**
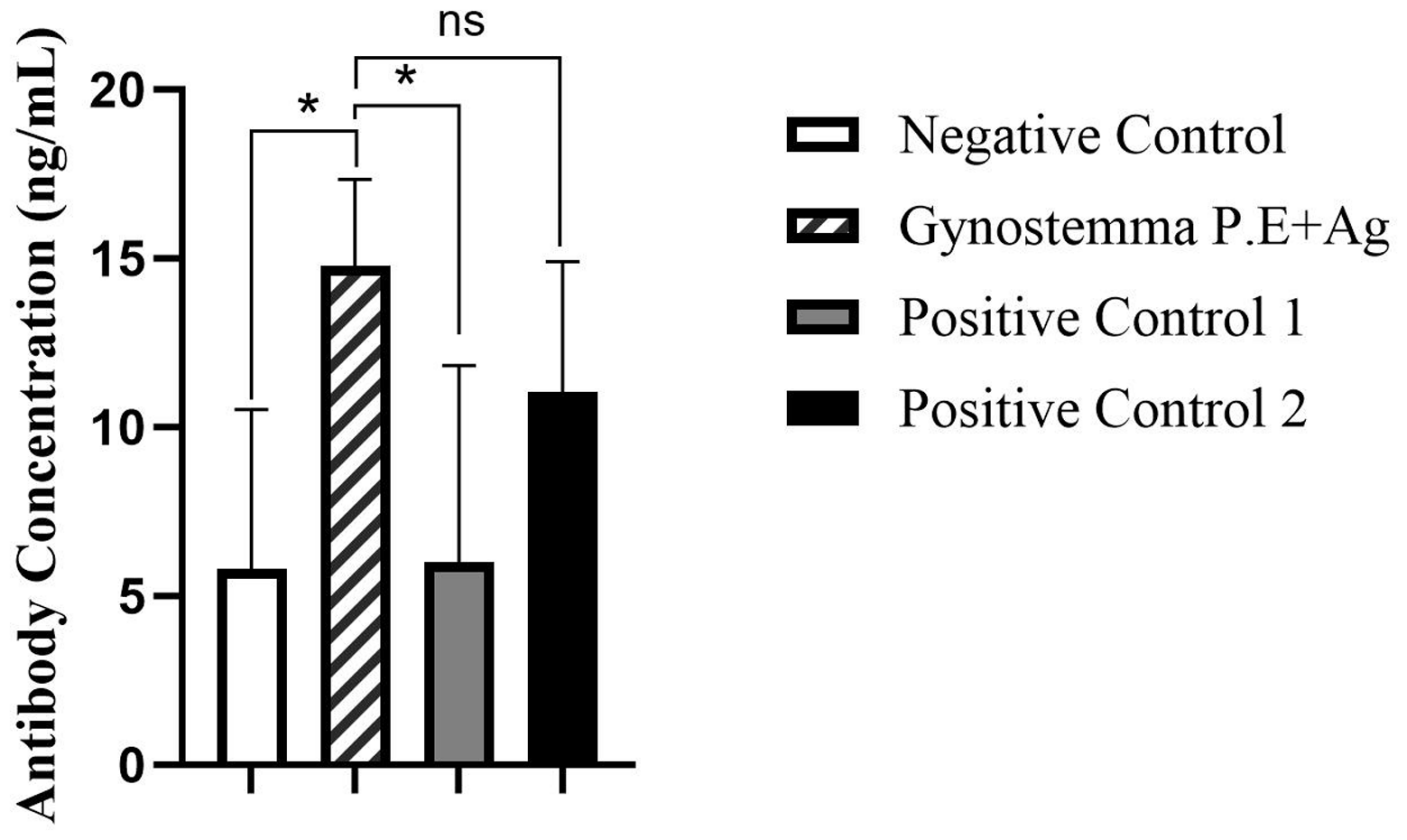
Measurement of IgA antibody levels in duodenal homogenate. * indicates a statistically significant difference between groups (*p* < 0.05); ns indicates no significant difference (*p* > 0.05).

As shown in Figure 1 serum IgG antibody levelsSerum IgG antibody levels, the serum IgG antibody levels in mice immunized intranasally with Gynostemma P.E combined with antigen were significantly higher than those in the antigen-only intranasal negative control group (*p* < 0.05), but showed no significant difference compared with the positive control groups (antigen injection or vaccine injection). Analysis of nasal lavage fluid IgA levels (Figure 2) revealed that the intranasal immunization group with Gynostemma P.E and antigen exhibited significantly elevated IgA titers compared to both the negative control group and the antigen injection group (*p* < 0.05), while no statistically significant difference was observed versus the vaccine injection group. Furthermore, duodenal mucosal supernatant IgA assays (Figure 3) demonstrated that the gypenoside-antigen intranasal group achieved markedly higher IgA antibody levels than the antigen-only intranasal control and antigen injection groups (*p* < 0.05), with no significant difference compared to the vaccine injection group.

### 3.2 Gynostemma P.E significantly enhanced splenic lymphocyte proliferation in mice

**Figure 4:**
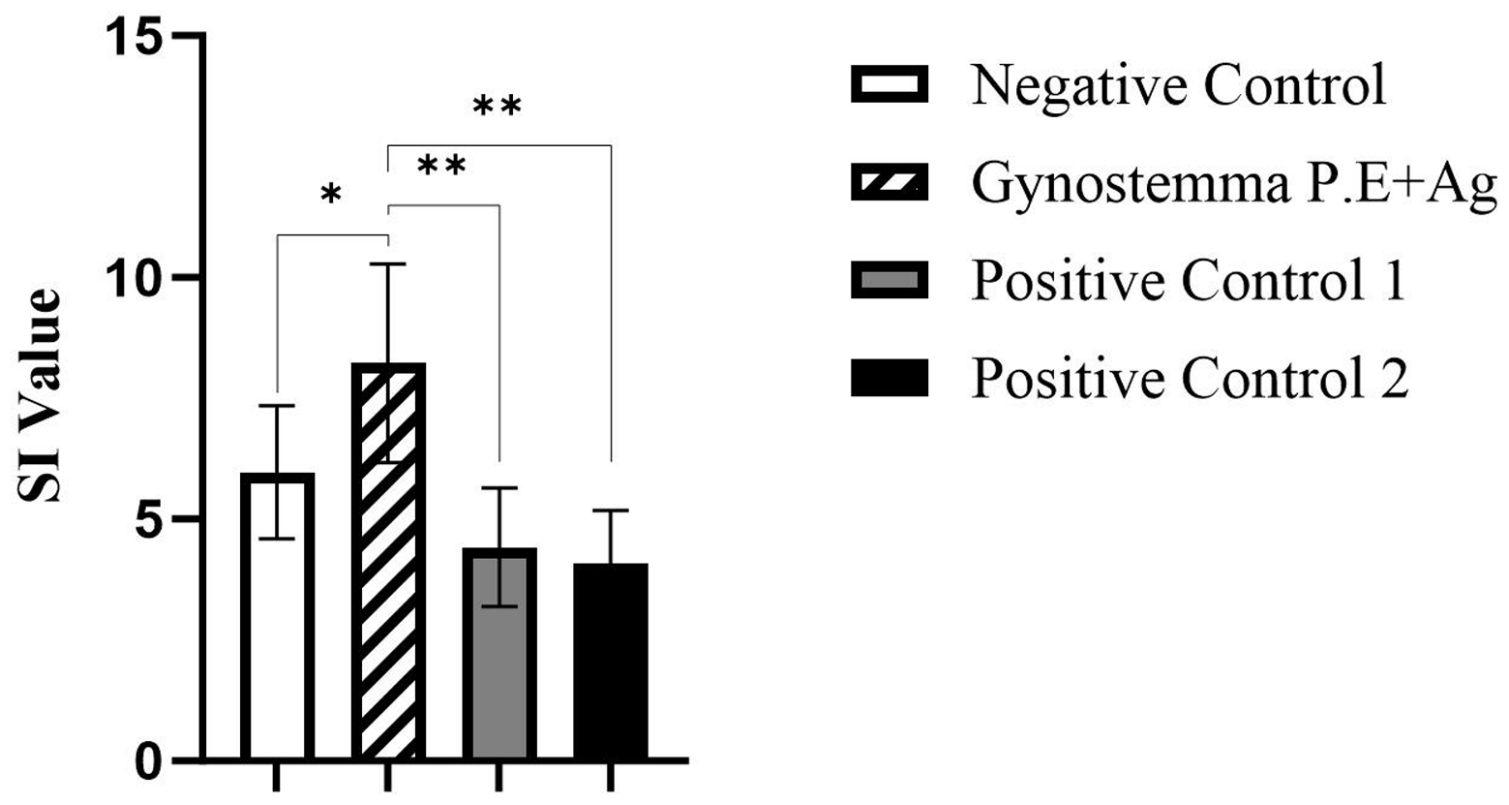
Effect of gypenoside on splenic lymphocyte stimulation index (SI). * indicates significant difference (*p* < 0.05); ** indicates highly significant difference (*p* < 0.01).

Two weeks after secondary immunization, spleens were aseptically harvested and lymphocyte proliferation was assessed by CCK-8 assay. The gypenoside plus intranasal antigen group exhibited a robust splenic lymphocyte proliferative response (Figure 4). Notably, the stimulation index (SI) in this group was significantly higher than that in both the antigen injection and vaccine injection positive control groups (*p* < 0.01). Moreover, compared with the intranasal antigen negative control group, the gypenoside plus antigen group showed a significantly increased SI value (*p < 0.05).

### 3.3 Higher intraepithelial lymphocytes (IELs) within the duodenum epithelium after gypenoside stimulation

**Figure 5:**
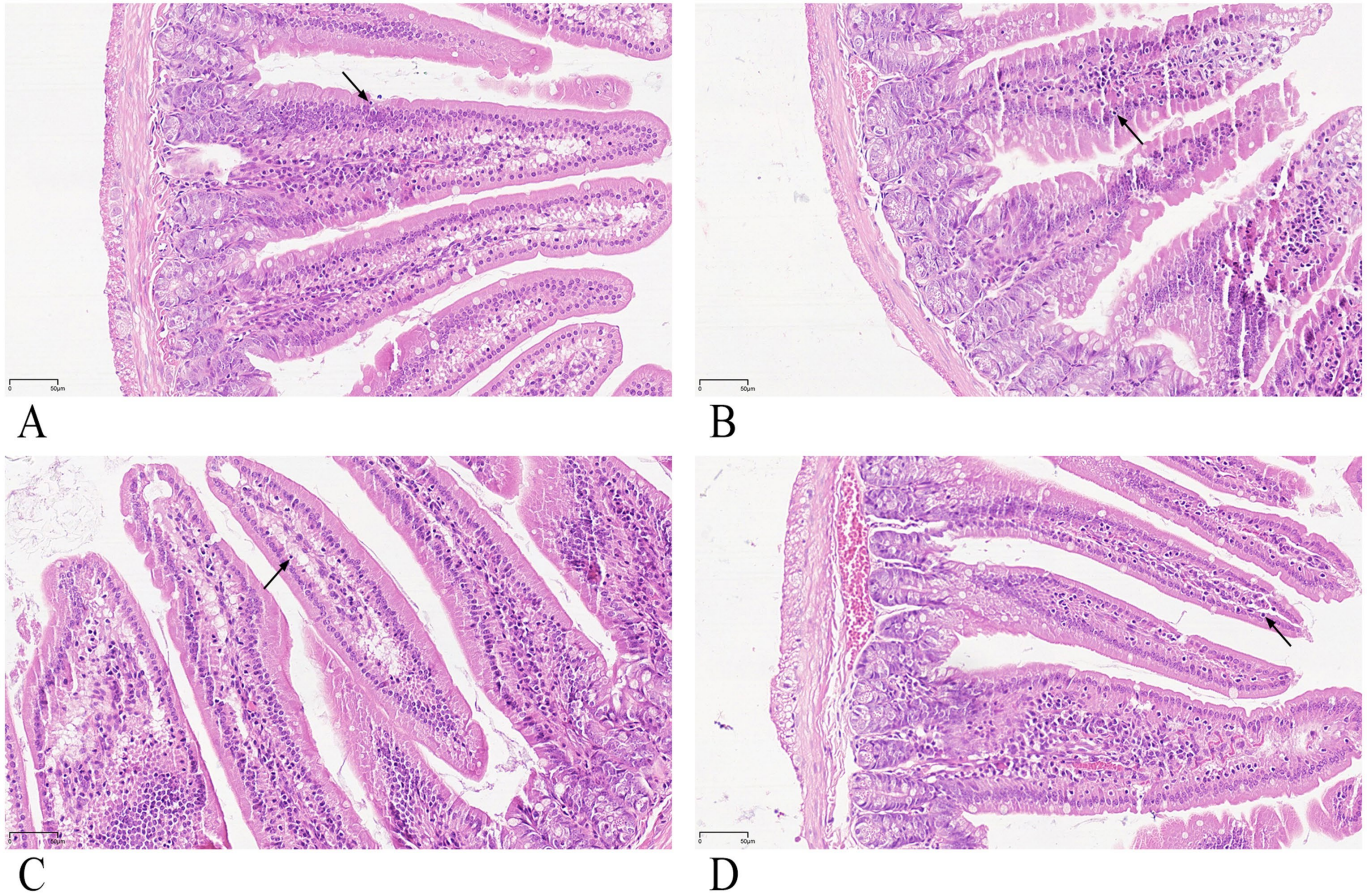
**(A–D)** Histological section of duodenum (H&E staining, 400×). **(A)** Intranasal antigen group (negative control). **(B)** Gynostemma P.E plus intranasal antigen group. **(C)** Antigen injection group (positive control 1). **(D)** Vaccine injection group (positive control 2). Black arrows indicate intraepithelial lymphocytes.

**Figure 6:**
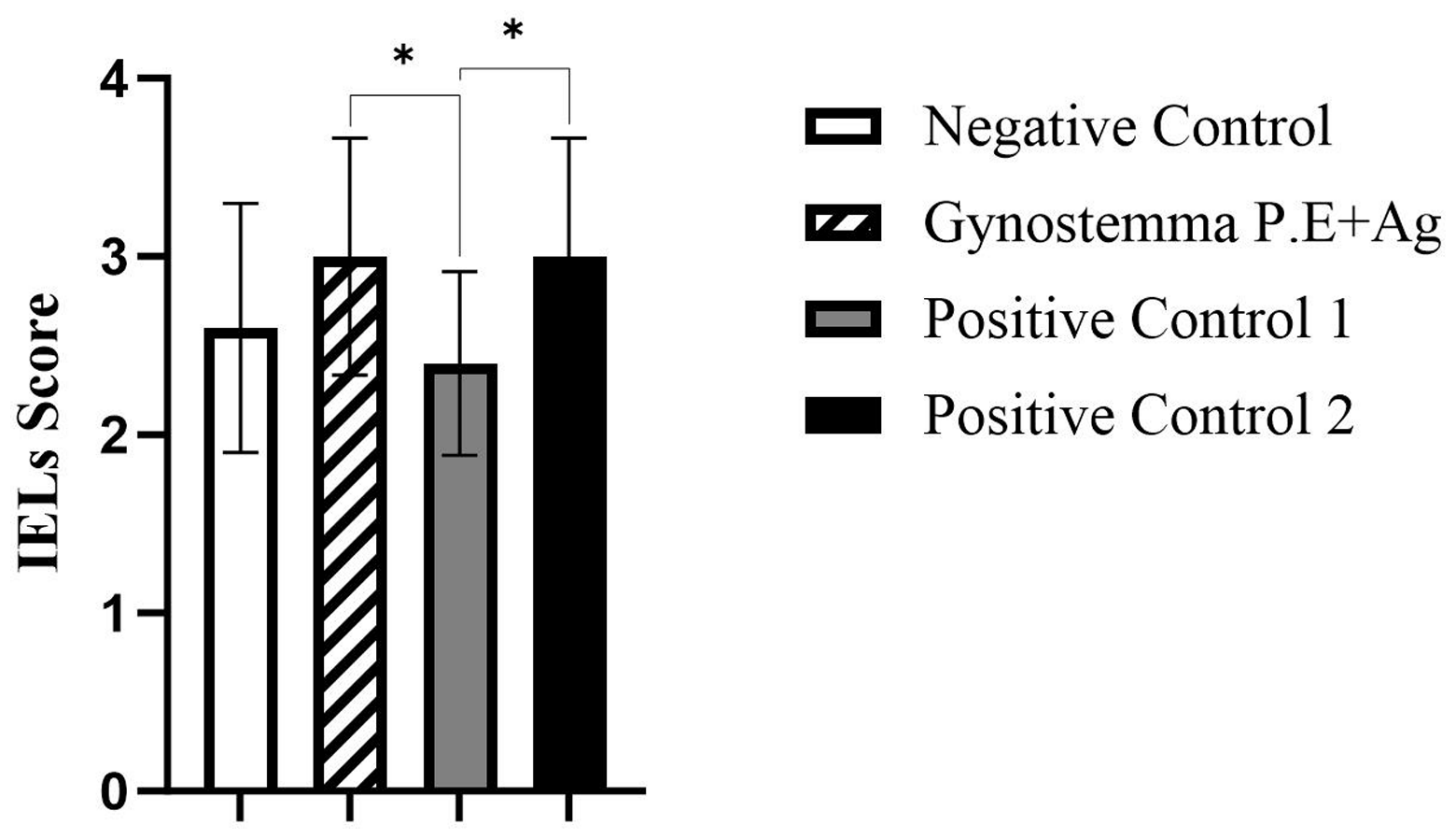
H&E staining analysis of IELs. * indicates a statistically significant difference between groups (*p* < 0.05).

H&E staining revealed round, densely stained nuclei (Figure 5, black arrows indicate IELs). Ten well-oriented intestinal villi per group were randomly selected for IEL counting. As shown in Figure 6, the gypenoside plus intranasal antigen group had significantly higher IEL counts than the antigen injection group (*p* < 0.05), but no significant differences were observed compared with the intranasal antigen group or vaccine injection group.

### 3.4 Gypenoside increased IgA-positive cells in intestinal mucosa

**Figure 7:**
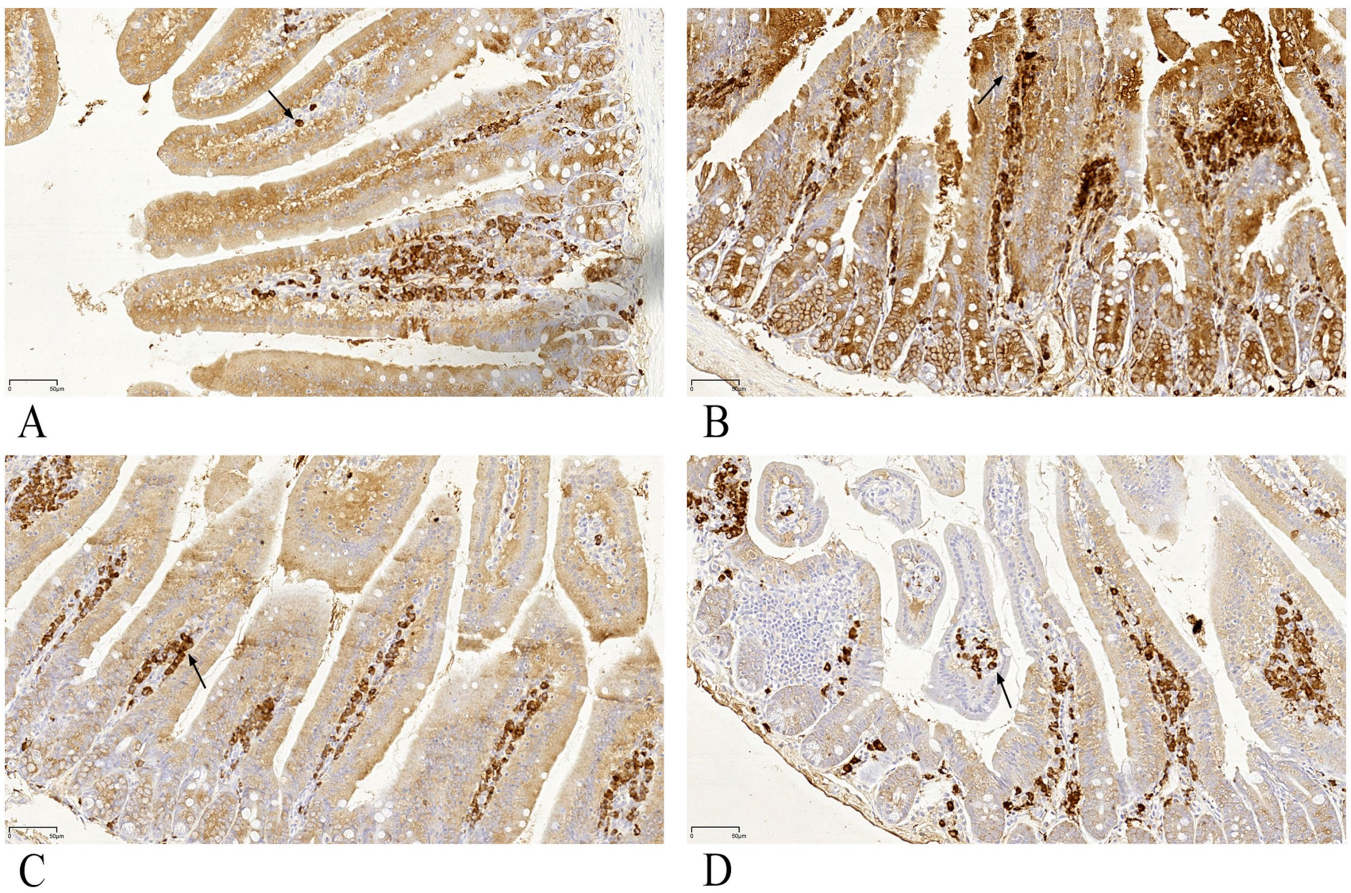
**(A–D)** Histological section of duodenum (immunohistochemical staining 400x). **(A)** Intranasal antigen group (negative control). **(B)** Gynostemma P.E plus intranasal antigen group. **(C)** Antigen injection group (positive control 1). **(D)** Vaccine injection group (positive control 2). Black arrows indicate intraepithelial lymphocytes.

**Figure 8:**
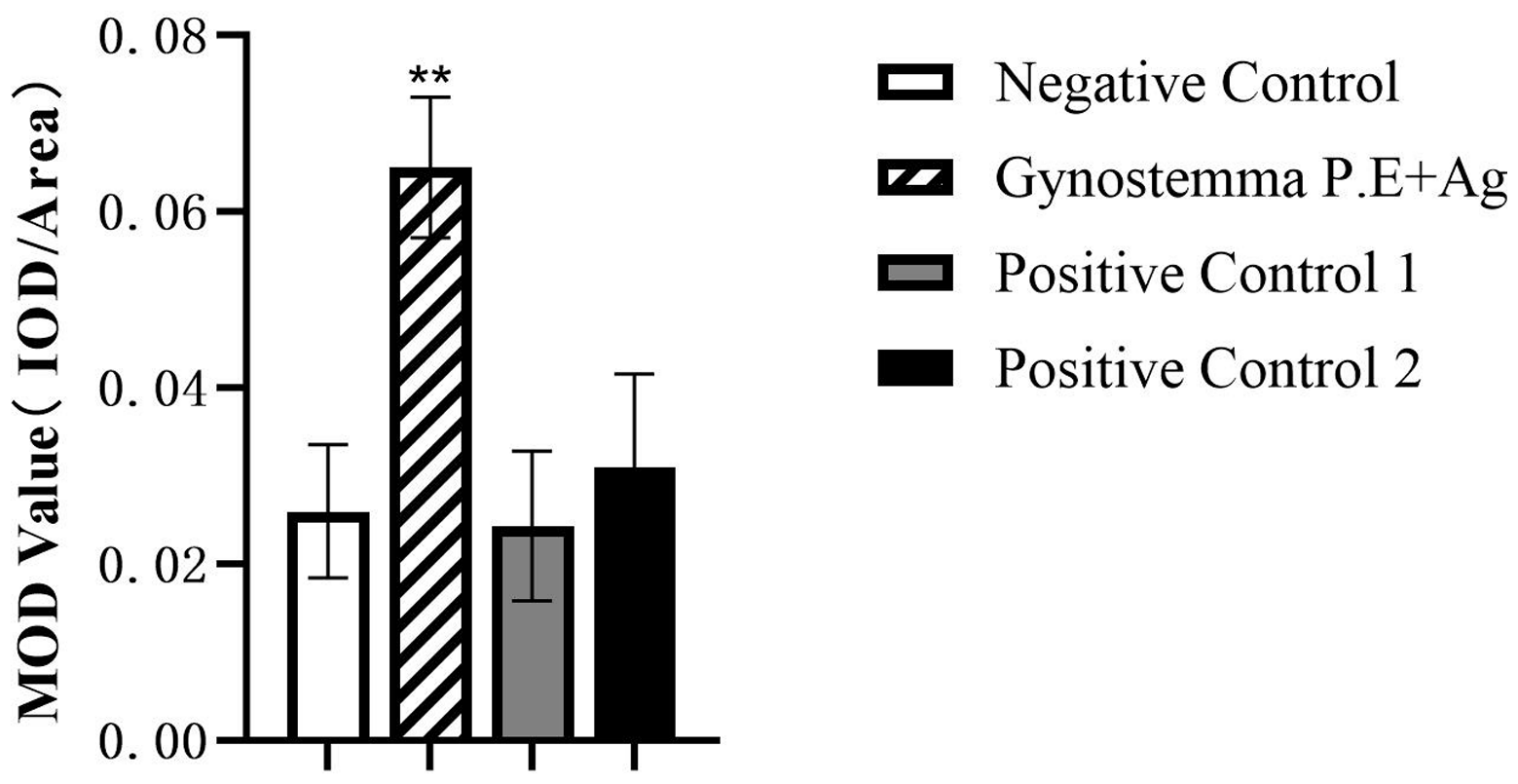
Immunohistochemical analysis of duodenum. ** indicates highly significant difference (*p* < 0.01).

IgA-positive cells (indicated by black arrows in Figure 7) were predominantly localized in the lamina propria of intestinal mucosa, with cytoplasmic brown staining. Semi-quantitative analysis using the MOD value (ratio of IOD to Area) revealed that the gypenoside plus intranasal antigen group exhibited a highly significant increase in IgA-positive cell numbers compared to the control group (*p* < 0.01, Figure 8).

## 4 Discussion

Porcine epidemic diarrhea (PED) is highly contagious and often breaks out successively in adjacent pig farms, causing catastrophic impacts on the region (13, 14). Studies report that mice inoculated with PEDV develop pathological changes including pulmonary inflammation and intestinal villous atrophy (15). Intramuscular injection of inactivated vaccine elevates antibody levels (16). Research demonstrated that intranasal inoculation of PEDV induces typical gastrointestinal symptoms in piglets. Compared to other routes, intranasal inoculation exhibits a longer incubation period, indicating viral transmission from nasal epithelium to the gastrointestinal tract (17). Current studies focus on antigen delivery mechanisms and intranasal immunization to enhance mucosal/systemic immunity (18, 19). Thus, intranasal inoculation serves as a reliable mucosal route for PEDV, further supporting airborne transmission. Additionally, as a key hub of mammalian mucosal immunity, intestinal epithelium acts as a real-time monitoring platform for systemic immunity, displaying distant infections (e.g., nasal mucosa) (20). Notably, PEDV primarily targets small intestinal epithelium. These collective findings provide the theoretical basis for this study’s use of nasal mucosal inoculation to enhance intestinal mucosal immunity.

This study evaluates the immunoenhancing effects of Gynostemma P.E on mucosal immunity against PEDV as a vaccine adjuvant. The experimental design followed the principle of equivalent inactivated antigen inoculation, and compared the efficacy differences among various immunization routes, as well as the effects of the extract on immune cell proliferation, specific antibody production, and intestinal mucosal immune responses. Groups A (negative control) and B (Gynostemma P.E + Ag) both received intranasal immunization, enabling direct comparison to reveal the immunoenhancement effect of Gynostemma P.E. Groups C (positive control 1) and D (positive control 2) both received intramuscular injection (representing conventional inactivated vaccination), allowing comparison to confirm ISA201 adjuvant efficacy. Groups A and C both received inactivated antigen alone (without adjuvants), permitting comparison of the intrinsic effects of the two immunization routes. Therefore, comparison between Groups B and D directly evaluates the novel GP-adjuvanted intranasal vaccine versus the current optimal conventional vaccination regimen (D Group). This design simultaneously validates both the adjuvant effect of Gynostemma P.E and the superiority of intranasal mucosal immunization.

In this study, the GP + antigen intranasal group showed significantly higher serum IgG levels than the negative control group (*p* < 0.05), higher nasal wash and duodenal homogenates IgA levels than all other groups except the vaccine injection group (p < 0.05). This indicates Gynostemma P.E can not only potentiate the immunogenicity of the intranasal route by activating mucosal immunity to promote local IgA secretion, but also significantly enhance systemic humoral immune responses and IgG production, thereby enhancing antigen-specific antibody responses. Although the precise mechanism requires further investigation, these findings provide important experimental evidence for the feasibility of needle-free immunization strategies.

The spleen as a critical immune organ contains abundant lymphoid tissue, including various immune cells. Upon initial antigen stimulation, antigen-presenting cells (APCs) present antigens and activate cellular and humoral immunity, while generating memory T cells and memory B cells. Upon re-exposure to the same antigen, these memory cells rapidly proliferate to achieve a swift immune response. Studies suggest that Gynostemma P.E enhance immune organ function by activating splenic lymphocyte proliferation or macrophage activation (21). As an early and easily quantifiable immune indicator, splenocyte proliferation exhibits high stability in cellular immunity evaluation and often correlates positively with final protective efficacy (22). To further understand the host’s immune status, this study measured splenocyte proliferation activity (SI value). Results demonstrated that intranasal co-administration of Gynostemma P.E and antigen significantly increased splenocyte proliferative activity compared to all control groups (*p* < 0.05). These results suggest that Gynostemma P.E may promote immune organ development, enhance T lymphocyte activation, and activate APCs or directly stimulate T cells to enter the cell cycle and proliferate. This process establishes the foundation for subsequent specific immune responses, thereby enhancing systemic immune competence.

Intraepithelial lymphocytes (IELs) and lamina propria lymphocytes in the small intestine constitute the effector sites of the gut mucosal immune system. IELs play a crucial role in mediating immune tolerance and regulation, demonstrating significant potential in immune surveillance (23–26). HE staining showed the number of duodenal IELs in the Gynostemma P.E + Ag intranasal group differed significantly only from the antigen injection group, suggesting Gynostemma P.E may enhance intestinal mucosal barrier function. The minimal difference from the vaccine injection group was possibly due to the complex functional interplay of IELs and the influence of adjuvants in the vaccine. Further research on cytokine production may clarify the reasons behind IEL population changes. IHC detection revealed significantly increased IgA + cell density (MOD value) in the lamina propria (*p* < 0.01), confirming that Gynostemma P.E promotes local immune cell recruitment and antibody secretion—potentially through regulation of cytokine networks like IL-5 and IL-6.

In conclusion, this study preliminarily demonstrates that Gynostemma P.E significantly enhances mucosal immunity induced by PEDV inactivated antigen while achieving comparable effects with higher safety. The results further clarify Gynostemma P.E’s immunoenhancing properties, providing a basis for its use in improving animal mucosal immunity, enhancing PED and other veterinary vaccines, and developing novel needle-free PED vaccine adjuvants. Future research is needed to focus on its active components and molecular mechanisms to facilitate clinical applications.

## Data Availability

The original contributions presented in the study are included in the article/supplementary material, further inquiries can be directed to the corresponding authors.
