# Correction: Immunoenhancing effects of Gynostemma Pentaphyllum Extract on mucosal immunity against porcine epidemic diarrhea virus

**DOI:** 10.3389/fvets.2026.1789645

**Published:** 2026-02-02

**Authors:** Yanxiang Zhang, Ruihan Tang, Yuanqing Liu, Zhihui Hao, Kai Fan, Shanshan Xie, Bo Tang, Shuaiyu Wang

**Affiliations:** 1 College of Veterinary Medicine, China Agricultural University, Beijing, China; 2 Agricultural College, Hubei Three Gorges Polytechnic, Yichang, China; 3 Innovation Center of Chinese Veterinary Medicine, China Agricultural University, Beijing, China; 4 Key Biology Laboratory of Chinese Veterinary Medicine, Ministry of Agriculture and Rural Affairs, Beijing, China; 5 College of Veterinary Medicine, Henan Agricultural University, Zhengzhou, China; 6 Beijing Biopharmaceutical Technology Center, Zhaofenghua Biotechnology Co., Ltd. (Nanjing), Beijing, China

**Keywords:** Gynostemma Pentaphyllum Extract, porcine epidemic diarrhea virus, mucosal immunity, antibody level, veterinary vaccine

The funder National Natural Science Foundation of China, grant no. 32172899, to China Agricultural University, SW, was erroneously omitted. The revised Funding statement reads:

“The author(s) declared that financial support was received for this work and/or its publication. This work was funded by the National Key Research and Development Program of China (grant no. 2022YFD1801101-2) (China Agricultural University, SW), the National Natural Science Foundation of China (grant no. 32172899) (China Agricultural University, SW), and the High-Level Talent Special Support Fund (grant no. 111/30501434) (Henan Agricultural University, SX).”

The original version of this article has been updated.

